# Mapping multiple components of malaria risk for improved targeting of elimination interventions

**DOI:** 10.1186/s12936-017-2106-3

**Published:** 2017-11-13

**Authors:** Justin M. Cohen, Arnaud Le Menach, Emilie Pothin, Thomas P. Eisele, Peter W. Gething, Philip A. Eckhoff, Bruno Moonen, Allan Schapira, David L. Smith

**Affiliations:** 1 Clinton Health Access Initiative, 383 Dorchester Ave., Suite 400, Boston, MA 02127 USA; 2 Swiss Tropical and Public Health Institute, Socinstrasse 57, 4051 Basel, Switzerland; 3 Center for Applied Malaria Research and Evaluation, Tulane University School of Public Health and Tropical Medicine, 1440 Canal St (2300), New Orleans, LA 70112 USA; 4 Oxford Big Data Institute, Li Ka Shing Centre for Health Information and Discovery, Nuffield Department of Medicine, University of Oxford, Oxford, OX3 7LF UK; 5 Institute for Disease Modeling, Building IV, 3150 139th Ave SE, Bellevue, WA 98005 USA; 6 Bill & Melinda Gates Foundation, PO Box 23350, Seattle, WA 98102 USA; 7 Independent Consultant, Legazpi City, Philippines; 8 Institute for Health Metrics and Evaluation, University of Washington, 2301 Fifth Ave., Suite 600, Seattle, WA 98121 USA

**Keywords:** Malaria, Risk mapping, Operational planning, Epidemiology, Health policy

## Abstract

There is a long history of considering the constituent components of malaria risk and the malaria transmission cycle via the use of mathematical models, yet strategic planning in endemic countries tends not to take full advantage of available disease intelligence to tailor interventions. National malaria programmes typically make operational decisions about where to implement vector control and surveillance activities based upon simple categorizations of annual parasite incidence. With technological advances, an enormous opportunity exists to better target specific malaria interventions to the places where they will have greatest impact by mapping and evaluating metrics related to a variety of risk components, each of which describes a different facet of the transmission cycle. Here, these components and their implications for operational decision-making are reviewed. For each component, related mappable malaria metrics are also described which may be measured and evaluated by malaria programmes seeking to better understand the determinants of malaria risk. Implementing tailored programmes based on knowledge of the heterogeneous distribution of the drivers of malaria transmission rather than only consideration of traditional metrics such as case incidence has the potential to result in substantial improvements in decision-making. As programmes improve their ability to prioritize their available tools to the places where evidence suggests they will be most effective, elimination aspirations may become increasingly feasible.

## Background

Past experiences with pathogen elimination underscore the importance of targeting the right tools to the right places [1, 2]. Attempting to achieve universally high coverage with an intervention in heterogeneous transmission environments may result in mismatched focus and effort: unnecessarily high coverage in places with minimal risk yet insufficient coverage in those focal areas where it would do the most good. This lesson was underscored during both the smallpox [1] and rinderpest [2] eradication campaigns, which achieved dramatic successes after shifting from mass vaccination to surveillance-driven targeting. The need for effective targeting is even greater for malaria programmes, since interventions such as indoor residual spraying of insecticides, insecticide-treated nets, and chemotherapy must be repeatedly deployed to maintain their effect. Targeting each tool to where it will be most effective while avoiding over-allocation of limited operational and financial resources [3] to unnecessary places [4] thus must become a high priority to maximize impact and sustainability.

The potential for mapping malaria transmission using relevant malaria metrics has grown in recent years, facilitated by the widespread availability of curated databases [5], geographic information systems, satellite imagery, and handheld tools for geocoded data collection and rapid reporting; model-based geostatistical frameworks; improvements in computational power; and household surveys with high-sensitivity diagnostics and standardized data management [5]. Maps of malaria prevalence [6–8], incidence [9–11], transmission events [12], intervention coverage [3], mortality [13], and “risk” defined in many different ways [14–19] have been published to help guide programmatic decision-making.

Although there is a long history of considering the constituent factors of malaria risk and the malaria transmission cycle via the use of mathematical models [20], and careful evaluation has been made of the comparative usefulness of a variety of metrics for measuring the impact of interventions [21], national strategic plans often make decisions about targeting interventions by relying upon categorizations of annual parasite incidence. These incidence measures alone describe only a single aspect of malaria risk: they cannot indicate whether transmission would persist or die out in the absence of importation [22], nor whether incidence is low only because good implementation of effective tools has reduced it from a much higher baseline [23], nor suggest what tools would be most impactful.

Basing decisions on more comprehensive assessments of transmission and its drivers can address these problems [14]. As in the parable of the blind men whose individual experiences of touching the trunk, the tail, or the legs of an elephant yield an incomplete picture of the entire animal, simultaneous consideration of multiple components of malaria risk can offer a more complete understanding of the transmission dynamics for operational planning. Measuring and mapping a broader set of malaria metrics related to these risk components has the potential to inform better geographical targeting of interventions and increase the impact of constrained resources.

## Malaria risk mapping in practice

A 2013 review by Omumbo et al. assessed the use of risk maps by national malaria control programmes across 47 countries in their national strategic plans and grant applications [24]. The review found that that nearly every country used maps for defining risk at sub-national levels. Although maps of malaria case incidence were the most common description of risk, used in about one-third of all maps, a range of other metrics including prevalence, qualitative descriptions, and climatic suitability were also mapped to describe malaria risk for programme planning.

Searching the published scientific literature reveals a similar variety of conceptions of risk mapping. A search for the terms “malaria”, “risk”, and “map” in the title or abstract of PubMed publications conducted on April 9, 2017 returned 134 publications. Of 110 of these which involved creation of malaria maps, the plurality (n = 46, 42%) involved mapping the prevalence of malaria infection, typically based on the results of a survey in which individuals were tested for the presence or absence of malaria infection. Other common types of risk maps included clinical case incidence (n = 21, 19%) and vector density or breeding sites (n = 14, 13%). Less commonly, risk was mapped according to estimated values of metrics such as the basic reproductive number R_0_ [25], the entomological inoculation rate (EIR) [15, 26, 27], and malariogenic potential [28] (the risk of malaria transmission, determined by importation and transmission potential [29]); Noor et al. mapped historical levels of prevalence as proxies for the baseline risk that would exist in the absence of control measures in Namibia [30], Somalia [31], and Sudan [32].

Together, these results suggest most decisions are being made on the basis of where malaria can be observed, whether on an incident basis via national surveillance systems or cross–sectionally through community prevalence surveys. Such practices align with historical elimination efforts, which were typically based on directing indoor residual spraying of insecticide campaigns according to maps of malaria prevalence as measured via population surveys [33].

**Fig. 1:**
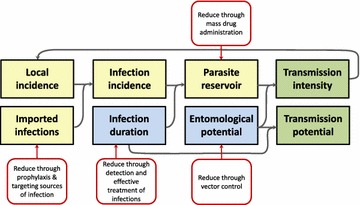
Components of malaria risk and relationships between them. Malaria risk can be considered as the combination of epidemiological factors typically measured programmatically (yellow boxes), factors influencing transmission rates (blue boxes), and measures of transmission potential or intensity (green boxes). Incident infections acquired both locally or imported replenish the parasite reservoir, with parasites persisting according to the human infection duration. The transmission intensity, which generates new local incidence, is determined by the combination of the human parasite reservoir and the entomological potential for transmission of that reservoir. Together, the mosquito-related entomological potential and the human-related infection duration largely comprise the transmission potential of a place, which describes the risk of malaria propagating there even if no parasites are currently circulating. Red boxes illustrate the interventions that reduce specific risk components

While accurate mapping of malaria incidence and prevalence yields important information for decision-making, malaria transmission intensity in a given place is the output of a dynamic process with multiple moving parts (Fig. 1) [34]. Observed infections may have been contracted locally or elsewhere, transmission intensity depends upon the interventions previously or currently implemented, and the causes of local incidence relate to both mosquito and human factors. Better operational efficiency might be achieved by considering not only the occurrence of malaria but also its component drivers, as described in Fig. 1, thereby identifying specific factors that may be particularly suitable for modification in certain places. Each of these risk components is now considered in turn, with description of its relevance for operational planning. A variety of metrics that may be measured to describe and map these components are also identified. Each of these metrics has associated measurement challenges and operational and financial costs, but offers programmes the opportunity to improve the tailoring and targeting of intervention packages for optimal impact of available resources. Metrics and operational implications are summarized in Table 1.

**Table 1:** Examples of how spatial overlay of multiple malaria metrics related to different components of malaria risk can inform elimination planning for a given operational unit

Malaria risk component	Proxy mappable metric	Information for decision-making	Example operational implication
Infection incidence	Annual parasite incidence	How much malaria is observed in the area?	Distribution of malaria drug demand; feasibility of implementing case-based surveillance
Local infection incidence	Local case incidence	Is transmission happening in the region?	Need for transmission intensity-reducing measures
Imported infections	Importation rate, human movement rate to endemic locations	Will new infections appear even if the local reservoir is drained?	Sustainability of aggressive reservoir reductions; need for importation-reducing measures
Parasite reservoir	Infection prevalence	Are infections currently available to be transmitted?	Potential for drug-based interventions to reduce transmission intensity by draining the available reservoir
Infection duration	Case treatment rate	How long will infections be available for mosquitoes to transmit?	Need for improved access to case management
Entomological potential	Vectorial capacity	What is the probability that parasites in the human reservoir will be transmitted?	Need for context-appropriate vector control measures
Transmission intensity	Entomological inoculation rate, ratio of local to imported cases	How much transmission is currently occurring?	Impact of currently implemented measures; need to examine specific risk components to identify appropriate additional measures
Transmission potential	Reproductive rates	Will introduced parasites tend to propagate or die away?	Need for reductions in infection duration or entomological potential to create conditions suitable for elimination

## Infection incidence

Infection incidence is the number of new infections that occur in a location per unit time. While the actual transmission event is not observable, subsequent clinical illness is, and the annual parasite index (API) thus is typically used as a proxy for infection incidence. This metric, which refers to the number of confirmed malaria cases identified per year, will be comprised both of infections transmitted locally and imported from elsewhere. API describes where malaria cases are observed, and thus where diagnosis and treatment commodities and services are required. In high endemic settings where most infections are locally acquired, API may also be an appropriate measure for describing malaria transmission risk, if the surveillance system is of good quality and provides high, continuous coverage. However, it is also possible to have a substantial API in the absence of transmission risk, since infections identified in urban centers, for example, due to importation of numerous infections from outlying areas or elsewhere abroad will usually be included in API even if these environments have little to no risk of transmission [35]. Even at a local level, transmission could occur at restricted times and places (e.g., logging camps [36]) but will be found later wherever humans live and are examined for parasites (e.g., at home, at school, or at a clinic). Every landscape is a heterogeneous patchwork, but since humans move further than mosquitoes, malaria will be found in more places than it is transmitted [34]. Although API may not be the best representation for transmission risk in such places, it may still be useful for describing the burden of malaria on the health system and may suggest where total incidence is sufficiently low that more intensive case-based surveillance measures may be feasible to implement [37].

## Local incidence

The incidence of locally-acquired infections is more relevant to directing interventions to places where they can minimize transmission than the incidence of all infections, since the latter includes imported infections that will not be affected by those interventions. Incidence of local clinical cases is typically measured and mapped [38] programmatically as a proxy metric for the incidence of local infections, though immunity and incomplete surveillance mean the incidence of infection and clinical malaria will not be equivalent. Places with the highest rates of local infection (or case) incidence are those where transmission intensity-reducing measures are most needed.

In low transmission settings, where there is a high probability that a given infection may have been acquired somewhere other than the location where it was observed, identified infections are typically classified according to whether or not the individual has a history of travel to known endemic regions within a biologically plausible time window, although specific methods for doing so vary between countries [39]. The confidence with which a case can be classified as local or imported based on a travel history will increase as transmission is reduced to very low levels, though travel histories may be incomplete or subject to recall biases [40, 41]. Genotyping provides one potential method for overcoming this gap, but such approaches are not yet practical (and have not been validated) for routine operations. Interventions act on local incidence by reducing other components of malaria risk that contribute to transmission intensity (Fig. 1).

## Imported infections

Quantifying the importation of infections into a region has long been recommended by the World Health Organization (WHO) as a means of classifying regions according to their “vulnerability” [42], or the risk of malaria being reintroduced following elimination efforts [29]. Imported malaria infections are typically ignored when local incidence is high, but importation will account for an increasing proportion of the parasite reservoir as local transmission declines and can fuel the reestablishment of transmission even if the local reservoir has been drained. For this reason, aggressive measures to eliminate the local reservoir must be accompanied by reduction in transmission potential sufficient to outweigh any importation that might otherwise re-establish transmission [43–45]. Understanding the heterogeneity in importation risk can inform operational planning by indicating where more aggressive elimination efforts may prove successful and where sustainable reductions in transmission will require ongoing efforts [46]. Since importation will be reduced when prevalence is reduced in the places from which imported cases originate, elimination planning that prioritizes high burden locations and/or coordinates elimination and control efforts nationally and across borders may also increase the efficiency of elimination efforts [18].

Importation can be represented by the rate at which infected humans or (less frequently) mosquitoes travel into a given place from elsewhere [12, 47, 48]. Proxy metrics for the importation rate can be calculated by direct measurement of imported malaria through surveillance. Accurate quantification of importation rates is challenging given case classification typically relies upon travel histories which may be incomplete or subject to recall biases, the existence of a travel history to an endemic area is not proof of infection there, and the fraction of imported (potentially asymptomatic) infections that are observed by the health system is likely incomplete [40, 41]. Alternatively, or in the absence of reliable data, the infection importation rate may be estimated by quantifying human movement to the place of interest from all other places with an existing parasite reservoir and adjusting by the probability of those humans being infected according to the prevalence in those places [47, 48]. Information on human mobility within [49] and between [50] countries can provide valuable information on importation risk. The importation rate into a given location is a dynamic quantity that can be reduced if malaria transmission is suppressed in connected populations, either by control activities or by providing prophylaxis to individuals traveling to at-risk locations.

At national level, imported case counts or rates refer to the number of observed cases that originated in other countries. Subnational maps of importation, however, should include all cases originating outside the operational unit in which they are diagnosed, regardless of whether the origin is within the country borders. If infections are imported in clearly defined high risk groups, prophylaxis or testing and treatment may be directed towards these individuals. Border screening of all travellers is used in some places but is unlikely to be cost efficient [51] for a number of reasons including the probability that travellers may be asymptomatic or have lower density infections that may be missed by conventional tests [52], especially for *Plasmodium vivax* malaria carriers who may only have undetectable hypnozoites. Chemoprophylaxis for travellers can reduce the risk of malaria in those moving between areas of no or low transmission and high transmission, but will only kill the hypnozoites of relapsing malarias if primaquine is included in the regimen. In many situations, it may be more practical to ensure good health services including surveillance for immigrants to minimize risk of transmission from imported infections rather than reducing importation directly.

## Parasite reservoir

The parasite reservoir comprises the pool of infected individuals in a place who could potentially transmit parasites to mosquitoes. It is quantified by the metric of infection prevalence, or the fraction of the population that is currently infected in an area. As with any disease prevalence, infection prevalence is the product of infection incidence and the duration of infections (described below). The shorter-lived population of infected mosquitoes that could transmit to humans also contribute to the parasite reservoir.

Population-based cross-sectional blood surveys can provide a point estimate of the overall prevalence in a target area, though the sample size to do so is typically infeasibly large in settings where prevalence may be lower than 1–3% [53]. In addition, mapping the variation in prevalence across a low endemic setting at high resolution is challenging given that parasites may be clustered, and any sample is likely to miss local heterogeneities. These gaps can be filled to some extent by making extrapolations from sampled population using geostatistical models and suites of covariates associated with infection [54]. The widespread availability of high-resolution, remotely-sensed imagery and data layers related to mosquito habitat and human populations provide data layers that can be used to correlate with observed patterns in malaria distributions [55].

Draining the parasite reservoir removes the infections available to be transmitted by mosquitoes, even if transmission-reducing measures are withdrawn. The parasite reservoir can be drained rapidly through mass drug administration, though in the absence of effective interventions to prevent reinfection, it is probable that this reduction will be short-lived in most settings [45]. Malaria transmission tends to be resilient, so that if the potential for transmission is not reduced, the reservoir will tend to recover after a sharp reduction unless there is nothing left to be transmitted, importation is low, and cases are rapidly detected and treated. Over time, the reservoir can also be drained through interventions that reduce transmission or infection duration, as described below.

## Infection duration

The parasite reservoir is replenished by incident infections and diminishes as human infections resolve, either through acquired immunity or rapid, effective treatment. Improved treatment rates can reduce transmission intensity by reducing the average duration of infections and thus decreasing the parasite reservoir available to be transmitted [56, 57]. The duration of an untreated infection will vary substantially by parasite species; studies of *Plasmodium falciparum* have suggested an average duration of 180–240 days, although infections lasting 300–400 days or longer have been observed [58]. Infectiousness waxes and wanes over the course of infections, however, and the highest probability of transmission may occur 3–10 weeks after infection [59]. As symptoms typically manifest around the tenth day of infection in non-immune individuals, prompt, effective treatment can greatly reduce the duration of infectivity of clinical cases [60]. However, in the presence of population immunity, most infections do not lead to symptoms, which is why elimination programmes are recommended to include extensive active case detection and sometimes other measures directed against parasite reservoirs.

Maps of treatment rates can provide a useful proxy measure for the duration of infections, a metric which may help programmes target improved case management or other drug-based strategies to places where they are most needed. The fraction of incident infections that receive treatment in a given place can be calculated as the product of a cascade of factors, including the fraction of all new infections that are symptomatic, that seek care, that are diagnosed with malaria, and that receive effective drugs. This fraction can be augmented through strengthened case management and case detection efforts, especially efforts that extend the reach of the health system into the community. Effective coverage of effective case management has been estimated to range from 7 to 71% across Africa [61]. Modelling of treatment rates in Tanzania suggested that replacing all anti-malarial drugs received at current treatment rates with effective artemisinin-based drugs might decrease malaria prevalence by between 21 and 53% [57]; increasing effective treatment rates by increasing the availability of effective drugs or actively looking for, testing, and treating malaria-positive fevers in the community would contribute to even greater reductions.

## Entomological potential

The probability that a given infection in the parasite reservoir will be transmitted on a given day is largely determined by the degree of human-vector contact. The entomological potential for transmission can be represented by the vectorial capacity, or the number of infectious bites that arise from each human infection per day that it is exposed to vectors. It refers to the effectiveness of a mosquito population in a given area at transmitting malaria, and is thus a metric for the entomological aspects of malaria risk.

Vectorial capacity is heavily influenced by ecological factors, including those of human ecology, such as housing quality and nighttime behaviours, as well as vector control strategies. It is not possible in practice to measure vectorial capacity across a region, but its spatial variation may be tentatively mapped according to entomological data and locally relevant ecological covariates which can be measured across the landscape [62]. Risk maps have also described patterns in components or correlates of vectorial capacity, including mosquito densities [63–65], breeding sites [66], and habitats for specific vectors [67, 68].

Since the entomological potential represents the mosquito-related components of transmission risk, it can be modified through implementation of vector control interventions, and places with greater potential (as represented by metrics such as the vectorial capacity) are those where context-appropriate vector control measures should likely be prioritized. Any intervention that reduces mosquito biting on humans, including nets, indoor spraying, attract and kill technologies, larval source management, environmental modifications, household improvements, or zooprophylaxis, will impact vectorial capacity. Choosing the most appropriate vector control measure in a given place requires further knowledge about vector biology, human ecology, and the preferences of the intended human beneficiaries.

## Transmission intensity

The combination of entomological potential and a parasite reservoir in a place determines the level of transmission intensity, or the amount of transmission that is currently occurring. Entomological potential can exist in places where there is no parasite reservoir [20, 69], and vice versa, and in such a place transmission intensity will be zero even though the individual risk components of entomological potential or parasite reservoir may exist. The greater the entomological potential and the more infections available to be transmitted, the greater the intensity of transmission that will result.

Transmission intensity is closely related to the force of infection [70], or the local incidence of new infections in the susceptible population. Transmission intensity in a place can be represented by the EIR, or the rate at which people are bitten by infectious mosquitoes [71, 72]. Mapping EIR requires measurements of its component factors, including mosquito density, biting rates, and the sporozoite rate in mosquitoes, and thus is uncommonly performed [26, 27]. However, simpler metrics that estimate transmission rates, including the ratio of local to imported cases at a given location [29, 73] and the size and/or longevity of outbreaks [74], may provide useful estimation of how transmission intensities vary across the region of interest. Analysis of genomic [75] or serologic [76] data may also estimate the transmission intensity. For example, simple models may be fit to measurements of seropositivity by age to assess the rate of transmission in the population [77].

Metrics related to transmission intensity can be used to assess whether the interventions being implemented are successfully reducing transmission rates [78]. Places with greatest transmission intensity are logically those where measures aimed at reducing it are likely most needed, though determining what interventions are most appropriate will require consideration of what specific factors are most responsible.

## Transmission potential

The potential for malaria transmission exists even if mosquito populations are currently being suppressed by vector control activities and/or if there are not currently parasites in that location to be transmitted. Transmission potential results from the machinery of the transmission cycle, including the presence of competent mosquito vectors (i.e., the entomological potential), and the probability that any infections that do exist in the location will remain uncured long enough to be picked up by those mosquitoes (i.e., the human infection duration). In other words, the transmission potential describes what transmission intensity would occur in the absence of interventions, assuming parasites exist or are introduced into that location.

If people rarely come into contact with competent mosquitoes (i.e., the entomological potential is very low) or nearly all incident infections are rapidly cured (i.e., the human infection duration is very short), then the transmission potential will also be low. The transmission potential that results from the combination of entomological potential and human infection duration in the absence of active interventions to modify them is classically represented by the basic reproductive number, R_0_ [79]. The R_0_ for a particular place describes the number of new infections that would arise from each existing infection in the absence of control measures, and its analogue R_C_ describes the current transmission potential given all the anti-malarial interventions applied at a given point in time.

If transmission potential can be maintained at a level at which each infection tends to lead to fewer than one new infection (i.e., R_C_ < 1), then malaria would eventually disappear in that place in the absence of importation of new infections, though it should likely be reduced to much lower levels (e.g., R_C_ < 0.5) to achieve elimination on rapid timeframes [80, 81]. A place where R_C_ is only somewhat less than one and where imported malaria is common may maintain a constant, substantial parasite reservoir and thus be difficult to distinguish from a place where R_C_ > 1.

Understanding heterogeneity in transmission potential is important for evaluating when ongoing control measures can be scaled back without risk of resurgence or reestablishment of transmission. Nevertheless, transmission potential is rarely mapped [25] directly [77], since it essentially involves measuring the unobservable counterfactual of what the malaria map look like in the absence of intervention. Somewhat complex mathematical modelling can be applied to derive measures of transmission potential from routinely available metrics such as infection prevalence [82], incidence [12], or combinations thereof [83]. Alternatively, the individual sub-components of transmission potential may be mapped individually, as described above. Another approach to mapping transmission potential is to describe the malaria risk that existed before interventions began; such an approach has been attempted in Botswana [84], Namibia [30], Somalia [31], Sudan [32], but its validity depends upon the degree to which intrinsic transmission risk has changed during the intervening years [85]. Related, serologic testing offers the potential to measure past exposure to malaria and thus to evaluate what transmission patterns might occur in the absence of intervention [86]. Transmission potential can be reduced through interventions resulting in structural or sustained changes to either its vector or human determinants, by ecological changes, and by health service improvements.

## Mapping multiple malaria metrics

The many components of malaria risk depicted in Fig. 1 means that maps of a single metric such as case incidence or infection prevalence will describe only a single aspect of risk and thus will be insufficient for optimal operational planning. Eliminating malaria requires some combination of draining the reservoir of infections (i.e., minimizing or entirely removing the presence of infections that might potentially lead to transmission) and preventing the transmission of any parasites that may remain or may be imported (i.e., reducing the transmission potential to near zero) [87]. In theory, elimination could be achieved in a place by reducing either of these components to zero without modifying the other; in practice, elimination is typically achieved by reducing both simultaneously, meaning that programmes need to simultaneously evaluate factors related to the parasite reservoir and those that determine its probability of being transmitted.

Efficient elimination planning thus involves understanding the distribution of multiple components of risk, including the relative contribution of imported and locally-acquired infections, the human duration of infection, the resulting parasite reservoir, the entomological potential, and the transmission potential that exists given the combination of infection duration and entomological potential. Overlaying maps of associated metrics, each of which constitutes a different type of malaria risk, supports strategic decision-making about what interventions will be most impactful in which places [88]. Consideration should also be given to how current distributions are influenced by the ongoing implementation of intervention measures.

Each risk component will be modified through specific sets of interventions, and each will typically exhibit considerable heterogeneity across the landscape in both its baseline and in the interventions implemented to reduce it. By mapping metrics representative of each of these sub-components of risk, it is thus possible to identify what package of interventions will be most effective at managing malaria risk in specific places. Places with high vectorial capacity will likely be the highest priority for measures to reduce human-mosquito contract, while those where infections have a long average duration require improved case management to cure more infections. However, the local characteristics of transmission must also be taken into consideration. If, for example, local vectors have bionomics constraining the effectiveness of available vector control options and these have already been implemented well, it may be rational to direct programme strengthening efforts towards reduction of infection longevity rather than desperately augmenting vector control.

Examples of how each of these components has been used to provide additional insights for operational planning beyond evaluation of incidence alone exist across a range of countries. Maps of metrics describing the parasite reservoir—where people are infected—illustrate the magnitude of malaria that remains and can demonstrate heterogeneity in reductions of malaria over time. For example, in Zambia, prevalence maps created from a series of infection prevalence surveys were used to assess the effect of bed nets over time, revealing significantly different impact in different parts of the country [89]. Maps of metrics related to the transmission potential—the degree to which any existing infections will reproduce—highlight areas of risk, even if current incidence levels are being suppressed by active intervention or after elimination has been achieved; for example, the likely prevalence if no interventions were implanted was mapped in Namibia to inform where interventions should be prioritized [30]. Locations with substantial imported malaria might require measures to reduce that importation, so routine surveillance data were used to map locations where importation occurred most frequently in Swaziland [12]. Aggressive measures to drain any local reservoir such as mass drug administration may be appropriate in places where importation rates are low and vectorial capacity and infection duration have already been reduced, as described in a modelling study considering variation in each of these factors [88].

Generic examples of how each of these risk components can inform programme decision-making about which interventions to deploy where are described in Table 1. Mapping metrics related to all risk components is not necessarily needed for planning, as measurement of each comes with an additional operational cost. Programmes will need to weigh the additional benefit of this information in terms of more tailored, targeted intervention planning against the operational and financial costs of collecting it.

## Discussion

A revolution in the availability of geolocated malaria data provides opportunities to achieve greater impact with today’s limited resources, yet as of today most strategic planning in endemic countries rarely takes full advantage of the available disease intelligence to tailor interventions. Decomposition of malaria risk into local determinants of transmission permits identification of specific interventions that will be most impactful in specific places, but the plurality of national malaria plans [24] focus on heterogeneity in malaria case incidence, while the published risk mapping literature primarily considers infection prevalence in the population. While incidence and prevalence are straightforward to collect through health information systems and population surveys, respectively, they are the products of several interacting factors which require explicit consideration in order to determine what interventions will be most effective in a given location.

While the WHO’s Global Technical Strategy aims for “universal” coverage with prevention, diagnosis, and treatment [90], budget realities require prioritizing certain interventions or packages of interventions, including efforts to strengthen testing and treatment, vector control such as distributions of nets or IRS, or mass or focal drug administration, to the places where they will be most effective. Elimination may require implementing multiple interventions in places with intensive focal transmission. Consideration of only the current amount of malaria across a region cannot clarify whether incidence or prevalence is suppressed from a greater intrinsic baseline, whether interventions can safely be removed without resurgence, or what the most appropriate interventions are to reduce transmission further. In addition, careful consideration of the movement of parasites between places can influence the staging of elimination strategies by prioritizing malaria reduction in source locations [91], potentially increasing cost-effectiveness [92].

This review describes a set of components that interact to comprise malaria risk. Measuring metrics related to components beyond only incidence and prevalence can give decision-makers a more complete picture of what interventions to implement where. Malaria transmission is the product of both human and vector factors, and given substantial heterogeneity in both across endemic regions, strategic planning may need to prioritize deployment of interventions that address specific components of the transmission cycle in places where they make particular contributions to transmission intensity and are particularly amenable to intervention.

In practice, it may be challenging to disentangle risk metrics given that available surveillance data will reflect their interaction. Collecting additional metrics comes at an operational and financial cost, and not all programmes will be able to measure all of the risk components described here. Furthermore, determining thresholds for categorizing maps of overlaid metrics into operational strata that can be assigned an optimal intervention package [93] will also require additional analysis. For example, determining whether importation is low enough to permit aggressive elimination to achieve sustainable elimination is a dynamic question that depends upon the strength of the health system to quickly identify and cure new infections as they occur, as well as the transmission potential in that location (e.g., even a low importation rate may cause problems in a place with high transmission potential, while higher importation may be absorbed in a place where transmission is quite low) [46]. Similarly, assessing whether vectorial capacity is sufficiently high or treatment rates sufficiently low to require intervention will not necessarily be self-evident. In many cases, mathematical modelling can be useful to help programmes make these decisions and optimize the allocation of their available resources [94].

Decision-makers will need to carefully consider the appropriate spatial scale for measuring the metrics described here. Although tools and resources exist for measuring each of these malaria metrics at extremely fine spatial scales, effective implementation of interventions requires generalizing to coarser operational units such as villages or districts. Given the increasing heterogeneity in malaria as transmission declines [95], it may be appropriate to initially map metrics at high resolution before aggregating to operational units at which interventions are to be conducted. Such an approach would ensure that focalized pockets of endemic transmission are not missed because of lower mean transmission rates across the broader areas.

This framework considers only general risk components (e.g., entomological potential) that may inform the need for broad categories of interventions (e.g., vector control), but further information and analysis will be required to identify what specific tools will be most impactful in which locations. For example, decisions as to where to use tools effective against indoor biting or resting mosquitoes versus other tools like larval control require additional stratification of entomological potential according to when and where biting or resting occurs. Similarly, decisions about how best to improve treatment rates must be context-adapted: interventions that focus on training healthcare workers at health facilities may be prioritized in places where the at-risk population has good access to those facilities (something that can be assessed using maps of treatment-seeking behaviour [96] and distance to health facilities [97]), while community health-workers may be superior strategies for increasing treatment rates in places that lack good access or where treatment-seeking rates are low.

## Conclusions

The widespread availability of geographic tools presents an important opportunity for malaria programmes to better stratify transmission intensity according to decision-relevant metrics. In doing so, it is important to recognize that many individual risks combine to drive transmission intensity, and accordingly multiple maps of different facets of malaria risk will be required to optimally plan responses. Implementing specific programmes based on knowledge of the drivers of malaria transmission in a location rather than only using metrics such as case incidence offers the potential to result in substantial improvements in decision-making. As programmes improve their ability to prioritize their available tools to the places where they will be most effective, elimination aspirations may become increasingly feasible.
